# The Impact of Body Mass Index Upon the Efficacy of Adalimumab in Hidradenitis Suppurativa

**DOI:** 10.3389/fmed.2021.603281

**Published:** 2021-06-22

**Authors:** John W. Frew, N. Singh, C. S. Jiang, R. Vaughan, J. G. Krueger

**Affiliations:** ^1^Laboratory of Investigative Dermatology, The Rockefeller University, New York, NY, United States; ^2^Department of Biostatistics, The Rockefeller University, New York, NY, United States

**Keywords:** hidradenitis suppurativa, acne inversa, adalimumab, body mass index, pharmacoepidemiogy

## Abstract

Elevated BMI in Hidradenitis Suppurativa is associated with decreased response to Adalimumab therapy. BMI is proposed to segregate distinct disease subtypes. It remains unresolved whether a threshold BMI exists above which increased dosages may provide clinical benefit. Individual patient data from 578 PIONEER Phase 3 participants were analyzed. Descriptive, multivariable regression analysis and receiver operating characteristic (ROC) curves were calculated to assess the relationship between BMI and clinical outcome measures using R v3.5.3. Participants in the overweight and obese BMI category had reduced odds (58 and 67%, respectively) of achieving HiSCR [OR = 0.42 (95%CI −0.19, 0.91) *p* = 0.03], [OR = 0.33 (95%CI 0.16, 0.67) *p* = 0.002] compared to participants with BMI < 25. Reduction in AN count and IHS4 score was not significantly associated. ROC analysis did not reveal any cut off value predictive of treatment outcome. No correlation between BMI and baseline disease activity or covariate interactions were identified. These findings suggest BMI is a significant covariate in the setting of lower baseline disease activity, supporting the concept of disease heterogeneity and differential therapeutic response to Adalimumab.

## Introduction

Hidradenitis Suppurativa (HS) is a chronic inflammatory disorder associated with obesity (1, 2) with increasing Body Mass Index (BMI) associated with increasing disease severity (3). Treatment guidelines for moderate to severe disease include the use of TNF-alpha antagonists (4) (Adalimumab) dosed at 40 mg every week in line with the pivotal PIONEER phase 3 clinical studies (5). Infliximab, also a TNF-alpha antagonist is used at a weight based dosage (6) (5 mg/kg/dose) in multiple inflammatory disorders, including psoriasis, rheumatoid arthritis and Crohn's disease, however increased disease control in HS is demonstrated at higher and more frequent dosages (up to 10 mg/kg every 6 weeks) (7). In Phase 2 studies the highest dosage administered was 40 mg per week and hence no data is available on the relative efficacy of doses >40 mg per week (8). Given that both Adalimumab and Infliximab are both TNF-alpha inhibitors, it is not unreasonable to assume that better disease control may be seen with increased dosages of Adalimumab particularly in patients with greater BMI. Additionally, our recent investigations have identified BMI as significantly associated with achieving clinical outcomes as measured by Hidradenitis Suppurativa Clinical Response (HiSCR), which is currently the gold-standard outcome for HS clinical trials (9). Other assessed clinical outcomes include change in abscess and nodule (AN) count, and the international hidradenitis suppurativa severity score (IHS4). As assessed using logistic regression, every unit increase in BMI significantly reduces the odds of achieving HiSCR by 7.1%. (OR = 0.93; 95% CI: 0.89, 0.97; *p* < 0.001). However, it is unclear whether this relationship is linear (as assumed above) or demonstrates increased significance above a certain threshold. Such relationships are assumed to be linear when analyzed by continuous variables, but their true relationship is only ascertained through more comprehensive analysis. It has also been reported that BMI may segregate distinct HS subtypes (10). Disease characteristics and comorbidities vary between HS patients with low (<30 mg/kg) and high (>35 mg/kg) BMI in case-control studies (10). Data supporting differential disease characteristics and response to therapy may support the premise that pathogenic heterogeneity exists, driving the search for more targeted individualized therapies in this often-recalcitrant disease.

Overall, the characteristics of the relationship between BMI and clinical response to Adalimumab in HS requires further detailed examination. Such statistical evaluation will enable us to accurately assess whether evidence exists for increased dosages of adalimumab beyond 40 mg per week, and for which patient populations this may be beneficial. Our overall aim is to characterize the relationships between BMI and clinical response (as measured by the HiSCR and IHS4) and investigate if a specific BMI cut-off can be identified which is associated with a reduction in clinical response. This would identify a patient subpopulation which may benefit from investigations into an increased dose of Adalimumab therapy.

## Materials and Methods

De-identified individual patient data (IPD) from the PIONEER 1 and PIONEER 2 Phase 3 studies of Adalimumab therapy in HS (5) were made available by AbbVie Inc. and accessed through the secure Vivli online platform. Raw data were extracted and compared to the available published data (5) to ensure accuracy. Only data for “Time Period A” (Week 0–Week 12) comparing Adalimumab 40 mg weekly vs. placebo was included in the analysis in order to reflect approved dosing regimens. Individuals with incomplete data and those who received antibiotic therapy in PIONEER 1 and those administered every-other-week (EOW) dosing were excluded from analysis. Antibiotic therapy in PIONEER 2 was included as a covariate. BMI was calculated as a continuous variable as well as a categorical variable in line with standardized BMI Categories (<25.0 kg/m^2^; 25.0 to <30.0 kg/m^2^; ≥30.0 kg/m^2^) (11) consistent with CDC and WHO recommendations. Due to the small number of subjects with underweight BMI (<18.5 kg/m^2^), this underweight category was merged with the normal BMI (18.5 to <25.0 kg/m^2^). All data analysis was conducted in R version 3.5.3 (12).

Each variable of interest was assessed for normality using the Shapiro-Wilk test and histograms. The differences between treatment groups were compared using Student's *t*-test for normally distributed continuous variables and the Mann-Whitney *U*-test for non-normally distributed continuous variables. Chi-squared and Fisher's exact-tests were used for categorical variables. Potential association of body mass index [BMI] variable with HiSCR response and IHS4 response, were assessed using Student's *t*-test for continuous BMI variable and Chi-squared-test for categorical BMI variables. Association of categorical BMI with absolute change in nodule counts and percentage change in IHS4 were assessed using one-way analysis of variance test (ANOVA) and *post-hoc* multiple comparisons tests were performed using Dunnett's method with underweight/normal BMI category as the reference group. Potential associations with categorical BMI, as well as other a priori potential associations [age, sex, Hurley stage, smoking status, family history, antibiotic use (PIONEER 2 only), and presence of draining tunnels] were assessed using logistic regression for HiSCR and binary IHS4 and linear regression for percentage change in IHS4 and absolute change in nodule count. Receiver operating characteristic (ROC) curve analysis was used for examining the best cutoff of BMI for predicting HiSCR and IHS4 response. Likelihood ratio tests and multiple partial F tests were performed to assess whether categorical BMI had a significant impact on the outcome of disease activity when adjusting for a priori covariates. The association of categorical BMI with absolute AN (abscess and nodule) count and IHS4 score was assessed using the Kruskal-Wallis H test. Spearman's rank order correlation analysis was performed to assess the correlation between continuous BMI and absolute disease activity variables. *P* < 0.05 was considered statistically significant.

## Results

For the purposes of our analysis, 144 and 145 individuals were included in the Adalimumab and placebo arms, respectively, of PIONEER 1; with 149 and 140 individuals included in the Adalimumab and Placebo arms, respectively, of PIONEER 2. The demographic and disease characteristics of these populations are included in Table 1. There was no statistically significant difference between the Adalimumab and placebo arms with regards to demographic and disease characteristics in both PIONEER 1 and PIONEER 2 (Table 1), although as previously reported (9) significant differences in race, median age, median BMI, nicotine use, median nodules and draining tunnels exist between PIONEER 1 and PIONEER 2 cohorts (9) (Supplementary Table 2).

**Table 1:** Characteristics of population in each of the trial data.

**Characteristic**	**PIONEER 1**			**PIONEER 2**		
	**Adalimumab**	**Placebo**	***P*-value**	**Adalimumab**	**Placebo**	***P-*value**
*N* =	144	145		149	140	
Female	85 (59.0%)	100 (69.0%)	0.10	97 (65.1%)	98 (70.0%)	0.45
Male	59 (41.0%)	45 (31.0%)		52 (34.9%)	42 (30.0%)	
White	111 (77.1%)	113 (77.9%)	0.35	130 (87.2%)	110 (78.6%)	0.07
Black	30 (20.8%)	25 (17.2%)		8 (5.4%)	18 (12.9%)	
Other	3 (2.1%)	7 (4.8%)		11 (7.4%)	12 (8.6%)	
Median age	35.0 (28.0, 45.0)	37.0 (30.0, 47.0)	0.14	35.0 (27.0, 42.0)	35.0 (26.0, 43.25)	0.49
Median BMI	32.1 (27.1, 38.0)	33.9 (28.5, 39.4)	0.07	30.3 (26.3, 36.0)	31.3 (26.8, 36.0)	0.22
**BMI category**						
Underweight/normal	24 (16.7%)	12 (8.3%)	0.09	32 (21.5%)	25 (17.9%)	0.43
Overweight	30 (20.8%)	36 (24.8%)		40 (26.8%)	32 (22.9%)	
Obese	90 (62.5%)	97 (66.9%)		77 (51.7%)	83 (59.3%)	
Hurley 2	80 (55.6%)	79 (54.5%)	0.95	76 (51.0%)	79 (56.4%)	0.42
Hurley 3	64 (44.4%)	66 (45.5%)		73 (49.0%)	61 (43.6%)	
Nicotine Use	77 (53.5%)	88 (60.7%)	0.26	96 (64.4%)	99 (70.7%)	0.31
Family History	37 (25.7%)	28 (19.3%)	0.25	36 (24.2%)	39 (27.9%)	0.56
Presence of Draining Tunnels	108 (75.0%)	108 (74.5%)	1.00	99 (66.4%)	87 (62.1%)	0.52
Antibiotics	–	–		27 (18.1%)	28 (20.0%)	0.80
Median nodules	8 (4.75, 14)	7 (4, 15)	0.88	6 (4, 11)	6 (4, 10.25)	0.98
Median abscesses	1.5 (0, 4)	2 (0, 3)	0.77	1 (0,3)	1 (0,3)	0.88
Median draining tunnels	2.5 (0.75, 7)	2 (0, 5)	0.38	2 (0, 4)	1 (0, 4)	0.60
Median baseline IHS4	26.5 (15, 45.25)	25 (12, 40)	0.28	19 (10, 34)	18 (8.75, 32.25)	0.91

No statistically significant association was identified between BMI (both as a continuous and a categorical variable) and participants achieving HiSCR in PIONEER 1 (Table 2). Similarly, no significant association was seen between BMI (both as a continuous and a categorical variable) and patients achieving IHS4 category change in PIONEER 1 (Table 2). A statistically significant association was seen between BMI as a continuous variable and achieving HiSCR (29.9 vs. 32.6, *p* < 0.001) and achieving IHS4 category change (30.0 vs. 32.2 *p* = 0.004) in PIONEER 2. This significant association also held when BMI was analyzed as a categorical variable against achieving HiSCR (*p* = 0.01) and IHS4 category change (*p* = 0.03; Table 2). The overall trend was for less patients achieving HiSCR or IHS4 category change with increasing BMI.

**Table 2:** (A) Association of BMI with treatment efficacy categorical variables and (B) Association of BMI with treatment efficacy continuous variables (Normal weight is the reference group).

**(A)**	**PIONEER 1**						**PIONEER 2**					
**BMI variable**	**Achieving HiSCR**		***P-*value**	**Achieving IHS4 category change**		***P-*value**	**Achieving HiSCR**		***P-*value**	**Achieving IHS4 category change**		***P-*value**
	Yes (*n* = 102)	No (*n* = 187)		Yes (*n* = 92)	No (*n* = 197)		Yes (*n* = 134)	No (*n* = 155)		Yes (*n* = 115)	No (*n* = 174)	
BMI (continuous)	33.9 ± 8.3	33.7 ± 7.7	0.84	35.0 ± 8.0	33.2 ± 7.8	0.07	29.9 ± 6.6	32.6 ± 6.4	** <0.001**	30.0 ± 6.3	32.2 ± 6.7	**0.005**
BMI underweight/normal	15 (41.7%)	21 (58.3%)	0.50	9 (25.0%)	27 (75.0%)	0.34	36 (63.2%)	21 (36.8%)	**0.01**	26 (45.6%)	31 (54.4%)	**0.03**
BMI overweight	20 (30.3%)	46 (69.7%)		18 (27.3%)	48 (72.7%)		33 (45.8%)	39 (54.2%)		36 (50.0%)	36 (50.0%)	
BMI obese	67 (35.8%)	120 (64.2%)		65 (34.8%)	122 (65.2%)		65 (40.6%)	95 (59.4%)		53 (33.1%)	107 (66.9%)	
**(B)**	**PIONEER 1**						**PIONEER 2**					
**BMI variable**	**%Change AN count**		***P*****-value**	**%Change IHS4 count**		***P*****-value**	**%Change AN count**		***P*****-value**	**%Change IHS4 count**		***P*****-value**
BMI underweight/normal	−36.2 ± 47.7		0.40	−25.8 ± 63.8		0.86	−51.2 ± 53.1		**0.02**	−43.2 ± 64.1		**0.04**
BMI overweight	−17.9 ± 63.9			−18.5 ± 64.0			−37.6 ± 59.1			−32.3 ± 60.5		
BMI obese	−23.2 ± 69.1			−21.6 ± 64.2			−25.4 ± 62.7			−17.7 ± 72.0		

*Bold value represents BMI, Body mass Index; HiSCR, Hidradenitis Suppurativa Clinical Response; IHS4, International Hidradenitis Suppurativa Scoring System*.

No significant association were identified between BMI categories and % change in AN count or % change in IHS4 count in PIONEER 1, but both comparisons were significant in PIONEER 2 (Table 2). *Post-hoc* Dunnett's-test between BMI categories and % change in AN count (obese vs. underweight/normal, *p* = 0.01) or % change in IHS4 count in Pioneer 2 (obese vs. underweight/normal, *p* = 0.03) showed significant difference between obese BMI category and underweight/normal BMI category. There was no difference between overweight vs. underweight/normal in *post-hoc* testing for the % change in AN count and % change in IHS4 count. Both the % change in AN count and % change in IHS4 count reduced with increasing BMI in PIONEER 2 whereas this trend was not seen in PIONEER 1 data (Table 2).

Logistic and Linear Regression Analysis examining BMI as categorical variables identified participants in the overweight and obese BMI categories as associated with decreased odds of achieving HiSCR compared to participants with BMI < 25 (Table 3). Participants in the overweight BMI Category had a reduction in the odds of achieving HiSCR of 58% [OR = 0.42 (95%CI 0.19, 0.91) *p* = 0.03] and participants in the obese category had a reduction in the odds of achieving HiSCR by 67% [OR = 0.33 (95%CI 0.16, 0.67) *p* = 0.002] compared to participants with BMI < 25 (Table 3). Categorical BMI demonstrated an association with achieving IHS4 category change; participants in the overweight BMI category had an increase in the odds of achieving IHS4 category change of 113% [OR = 2.13 (95% CI 1.16, 3.95) *p* = 0.02] as compared to obese BMI category. Overweight BMI was also significant as assessed using the likelihood ratio test.

**Table 3:** Results of logistic regression models of HiSCR achievement (Model 1) and IHS4 category change (Model 2) in patients treated with Adalimumab and Placebo in PIONEER 1 and PIONEER 2.

**Variable**	**PIONEER 1 Achieving HiSCR**			**PIONEER 2 Achieving HiSCR**		
**Model 1**	**Odds ratio**	**95 % CI**	***P-*value**	**Odds ratio**	**95 % CI**	***P-*value**
Adalimumab	**2.02**	**(1.22, 3.38)**	**0.007**	**4.20**	**(2.50, 7.24)**	** <0.001**
Hurley stage 3	0.90	(0.51, 1.57)	0.70	0.63	(0.36, 1.09)	0.10
Family history	0.76	(0.41, 1.39)	0.39	0.70	(0.38, 1.27)	0.24
Current smoker	0.98	(0.59, 1.65)	0.95	0.57	(0.32, 1.01)	0.06
Presence of draining tunnels	0.65	(0.34, 1.21)	0.17	**0.49**	**(0.27, 0.87)**	**0.02**
Antibiotic use	–	–	–	**0.45**	**(0.22, 0.90)**	**0.03**
BMI (overweight)	0.74	(0.31, 1.78)	0.49	**0.42**	**(0.19, 0.91)**	**0.03**
BMI (obese)	0.88	(0.41, 1.92)	0.75	**0.33**	**(0.16, 0.67)**	**0.002**
Male sex	0.87	(0.50, 1.51)	0.62	0.98	(0.54, 1.77)	0.95
Age	1.00	(0.97, 1.02)	0.74	1.00	(0.98, 1.02)	0.95
**Variable**	**PIONEER 1 achieving IHS4 category change**			**PIONEER 2 achieving IHS4 category change**		
**Model 2**	**Odds ratio**	**95 % CI**	***P-*****value**	**Odds ratio**	**95 % CI**	***P-*****value**
Adalimumab	**1.67**	**(0.99, 2.83)**	**0.05**	**2.89**	**(1.74, 4.88)**	** <0.001**
Hurley stage 3	**0.51**	**(0.29, 0.86)**	**0.01**	**0.57**	**(0.33, 0.95)**	**0.03**
Family history	1.01	(0.54, 1.86)	0.96	1.28	(0.72, 2.28)	0.40
Current smoker	0.79	(0.46, 1.33)	0.37	1.03	(0.60, 1.80)	0.91
Antibiotic use	–	–	–	0.72	(0.36, 1.38)	0.32
BMI (overweight)	1.35	(0.52, 3.67)	0.54	1.30	(0.62, 2.75)	0.49
BMI (obese)	1.50	(0.66, 3.67)	0.34	0.63	(0.32, 1.22)	0.17
Male sex	0.72	(0.40, 1.27)	0.26	0.58	(0.33, 1.02)	0.06
Age	1.00	(0.98, 1.02)	0.99	0.99	(0.97, 1.02)	0.64

*Bold value represents BMI, Body mass Index; HiSCR, Hidradenitis Suppurativa Clinical Response; IHS4, International Hidradenitis Suppurativa Scoring System*.

In analysis with both categorical and continuous BMI there was no significant association with change in AN count (Table 4). However, in percentage change in IHS4 score, each unit increase in BMI (as a continuous variable) attenuated the percentage reduction in IHS4 score by 1.65% (*b* = 1.65; 95% CI: 0.50, 2.81; *p* = 0.01). The multiple partial *F*-test suggested that categorical BMI did not have a significant effect on the percentage change in IHS4 when adjusting for covariates.

**Table 4:** Linear regression model of change in AN count (Model 1) and % change in IHS4 outcome measure in (Model 2) in Adalimumab treated patients in PIONEER 1 and PIONEER 2.

**Variable**	**PIONEER 1 change in an count**			**PIONEER 2 change in an count**		
**Model 1**	**Estimate**	**95 % CI**	***P-*value**	**Estimate**	**95 % CI**	***P-*value**
Adalimumab	**−2.26**	**(−4.31**, **−0.21)**	**0.03**	**−2.56**	**(−3.95**, **−1.16)**	** <0.001**
Hurley stage 3	**2.36**	**(0.12, 4.60)**	**0.04**	−0.18	(−1.64, 1.28)	0.81
Family history	−0.73	(−3.19, 1.73)	0.56	−0.58	(−2.17, 1.01)	0.47
Current smoker	−0.88	(−2.97, 1.21)	0.41	−0.02	(−1.54, 1.49)	0.98
Presence of draining tunnels	1.02	(−1.57, 3.61)	0.44	**1.88**	**(0.32, 3.44)**	**0.02**
Antibiotic use	–	–	–	1.10	(−0.68, 2.87)	0.22
BMI (overweight)	0.44	(−3.14, 4.02)	0.81	−0.13	(−2.22, 1.96)	0.90
BMI (obese)	0.21	(−2.97, 3.39)	0.90	0.11	(−1.73, 1.95)	0.91
Male sex	−0.41	(−2.65, 1.83)	0.72	0.05	(−1.52, 1.62)	0.95
Age	0.03	(−0.06, 0.12)	0.55	0.03	(−0.04, 0.09)	0.40
**Variable**	**PIONEER 1 % change in IHS4**			**PIONEER 2 % change in IHS4**		
**Model 2**	**Estimate**	**95 % CI**	***P-*****value**	**Estimate**	**95 % CI**	***P-*****value**
Adalimumab	–**18.70**	**(**–**33.81**, –**3.58)**	**0.02**	–**39.94**	**(**–**55.17**, –**24.71)**	** <0.001**
Hurley stage 3	8.31	(−6.99, 23.60)	0.29	2.79	(−12.67, 18.25)	0.72
Family history	−3.67	(−21.62, 14.28)	0.69	−5.97	(−23.35, 11.41)	0.50
Current smoker	1.13	(−14.22, 16.49)	0.88	4.75	(−11.78, 21.29)	0.57
Antibiotic use	–	–	–	18.14	(−1.22, 37.50)	0.07
BMI (overweight)	1.13	(−25.17, 27.43)	0.93	9.49	(−13.35, 32.33)	0.41
BMI (obese)	2.88	(−20.42, 26.17)	0.81	**22.61**	**(2.48, 42.75)**	**0.03**
Male sex	10.56	(−5.79, 26.92)	0.20	6.79	(−9.72, 23.30)	0.42
Age	0.28	(−0.39, 0.94)	0.42	−0.07	(−0.76, 0.62)	0.84

*Bold value represents BMI, Body mass Index; HiSCR, Hidradenitis Suppurativa Clinical Response; IHS4, International Hidradenitis Suppurativa Scoring System*.

### Receiver Operating Characteristic Curve Analysis

**Figure 1:**
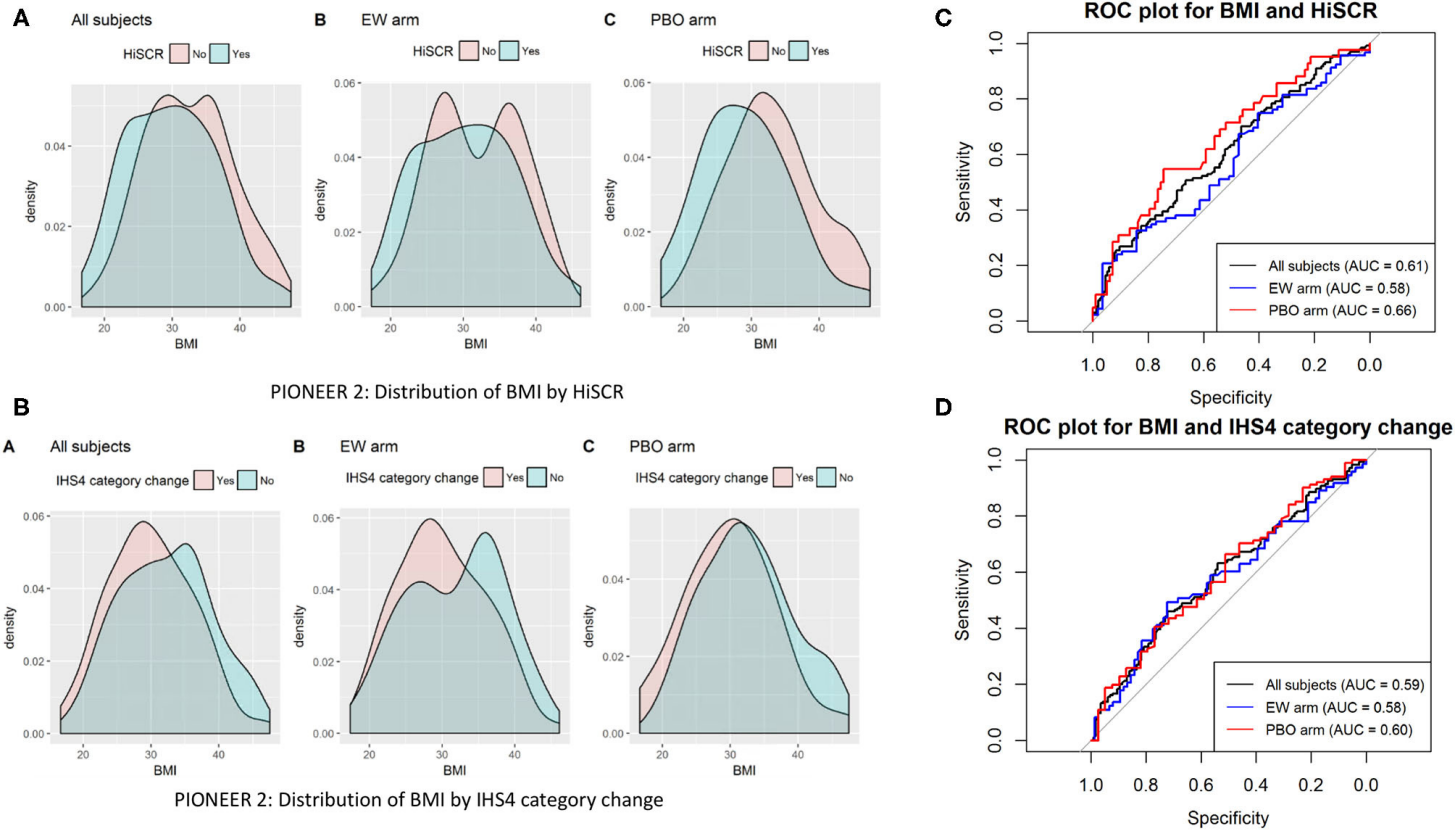
Distribution **(A,B)** and ROC plots **(C,D)**. Receiver operating characteristic (ROC) curves of BMI against HiSCR and IHS4 category change.

Given the discrepancies between continuous and categorical BMI, we enquired as to whether a specific BMI could be identified as the point with the most appropriate cut off for specific measurements of disease activity (HiSCR and IHS4). Through ROC analysis (Figure 1), no specific cut off was identified with high sensitivity and specificity for predicting HiSCR and IHS4 response in PIONEER 2 (the only study in which BMI was a significant covariate). The area under the ROC curve (AUC) was poor in the analysis with all subjects and by treatment arm (0.58–0.66).

## Discussion

HS is a heterogenous disease (13, 14), but the relationship between this heterogeneity and BMI is incompletely defined. BMI is significantly associated with achieving HiSCR regardless of the status of BMI as a categorical or continuous variable. The reduction in odds is greater for participants in the obese category than the overweight category suggesting the accuracy of the general assumption that increasing BMI pre-disposes to a decrease in the efficacy of Adalimumab as measured by achieving HiSCR (9). BMI was significantly associated with change in AN count in univariate analysis (Table 2) in Pioneer 2 but was not significant when other variables including aspects of disease severity were taken into account (Table 4). It appears that the significant association of BMI in clinical response to Adalimumab is restricted to outcomes assessing a proportional rather than an absolute change in disease activity. This suggests that the association may be a product of the outcome measure rather than a true association; and may be more likely in those with lower baseline disease activity given that proportional change is inherently greater in the setting of lower baseline values.

This hypothesis would agree with what is observed with the difference in regression analyses between the PIONEER 1 and 2 studies (Tables 3, 4). Disease severity (as measured by AN count and draining tunnel count) is significantly greater in PIONEER 1 than PIONEER 2 (Supplementary Table 1). Additionally, the median BMI for the PIONEER 1 cohort was significantly higher than the median BMI for the PIONEER 2 cohort (32.5 vs. 31.2 *p* < 0.001 by the Mann-Whitney *U*-Test) (Supplementary Table 1). This difference in BMI is equivalent to a 4% reduction in absolute weight (given an unchanging height) which is reported within the range of clinically significant weight change (15). In order to explore these observations further we examined the correlation between BMI and baseline disease activity (as measured by number of nodules/abscesses/AN count/number of draining tunnels) (Supplementary Figure 1). These results supported the findings of previous reports (2, 3, 16, 17) that BMI is not significantly associated with baseline number of nodules/abscesses/AN count/number of draining tunnels/IHS4 score although it is related to other outcomes such as the Sartorius score (16, 17). BMI accounts for <2.89% of the variation in baseline disease activity in PIONEER 2 across all measured examined (Supplementary Figure 1).

If significant correlation between BMI and baseline disease activity did exist, the significant findings in % change scores (HiSCR and % IHS4) compared to absolute changes (AN count and IHS4 category change) are logical. For a given dose of medication it is harder to achieve the same percentage change from a higher baseline than it is from a lower baseline. However, given the presented data, one must conclude that an alternate reason for the discrepancy exists. The lack of association of BMI with disease activity and treatment response in PIONEER 1 as opposed to PIONEER 2 (Tables 3, 4) raises the prospect that BMI may only demonstrate a significant association with response to Adalimumab in a subpopulation of participants with HS. This supports the previously reported concept of disease and pathogenic heterogeneity in HS (2, 17). Given the differences between the two PIONEER studies it may be hypothesized that the characteristics of the PIONEER 2 cohort are more reflective of the subpopulation (moderate disease, primarily nodules, low number of draining tunnels) where BMI demonstrates a significant association with clinical response to Adalimumab therapy.

In order to investigate this hypothesis, we analyzed the interaction between draining tunnels and BMI (both being significant covariates) as well as the contribution of baseline AN count in the linear and logistic regression models previously presented (Tables 3, 4). No significant interaction effect was noted between BMI and the presence or absence of draining tunnels. However, when baseline AN count was introduced as a covariate it was (as expected) significantly associated with absolute change in AN count [*b* = −0.37 (95%CI = −0.44, −0.30) *p* < 0.0001] and IHS4 Category change [OR = 0.94 (95%CI = 0.90, 0.97), *p* = 0.002] in PIONEER 2. The significance of BMI was not altered with the addition of the baseline AN covariate into the regression model, although Hurley stage became non-significant in the logistic regression model of achieving IHS4 category change (Supplementary Table 2). One critique of examining BMI by categorical variables is that traditional BMI cut-offs are assumed to be best suited to understanding the relationship between BMI and HS disease severity scores. However, in order to not rely upon this assumption, we examined this relationship using ROC, sensitivity and specificity analyses, and no alternative cut off was identified to further examine this relationship.

Overall, these in-depth analyses suggest that the significant association of BMI in the response to Adalimumab is maintained when other factors of baseline disease activity are taken into account. This supports the concept that BMI plays a significant independent role in disease response to Adalimumab therapy, however this significance may only be present in a subpopulation of individuals with HS given the discrepancies between regression models and PIONEER 1 and 2 cohort characteristics.

The findings of this analysis can be used to direct further mechanistic enquiry into the molecular pathogenesis of HS. Adipose tissue derived pro-inflammatory mediators (such as polyunsaturated fatty acids and the lipoxygenase pathway) (18) have been demonstrated to be significantly elevated in lesional HS tissue (nodules) as well as in individuals with elevated BMI (19) compared to healthy controls (18). These observations suggest that such mediators may have a role in the initiation phase (20) of clinical disease in HS (associated with reduced number of lesions) rather than activity in severe established disease. This hypothesis would be supported by the evidence surrounding bariatric surgery and weight loss in the treatment of HS (21–23), with reports of excellent response only in Hurley stage 1 and 2 patients (21–23).

Limitations to this study include the inherent limitations of using clinical trial data, including acknowledging the limitations in extrapolating this data to the wider HS population (24).

Based upon the body weights of participants in this study, the range of dosages (mg/kg) for Adalimumab only ranged between 0.26 and 0.93 mg/kg and hence any extrapolation beyond this range of dosages is inaccurate. In addition, the potential mechanistic links between BMI and nodules require further investigations in molecular, mechanistic and translational studies.

BMI is significantly associated with response to Adalimumab in the treatment of HS as measured by HiSCR. An increase in BMI was associated with decreased odds of achieving HiSCR in the PIONEER 2 study. BMI was not significantly associated with IHS4 category change or change in AN count; but percentage change in IHS4 score was significantly attenuated in participants with obese BMI. The discrepancies between the association of BMI and treatment response may be explained by BMI only being a significant influence in a subpopulation of participants with lower baseline AN count. Therefore, further mechanistic studies are needed to reliably identify this subpopulation in which BMI has a significant association with response to Adalimumab and evaluate the role of increases doses of Adalimumab or weight loss interventions in this cohort.

## Data Availability Statement

The data analyzed in this study is subject to the following licenses/restrictions: This publication is based upon research data from Abbvie Inc. Abbvie Inc. had no input into the design or execution of the study, statistical analysis or composition of the article. Requests to access these datasets should be directed to Vivli Inc. support@vivli.org.

## Ethics Statement

The studies involving human participants were reviewed and approved by this study is a re-analysis of de-identified patient data provided by data contributor Abbvie Inc. through Vivli Inc. This re-analysis was approved by the Institutional Review Board of the Rockefeller University. The patients/participants provided their written informed consent to participate in this study.

## Author Contributions

JF and RV designed the study. JF, NS, and CJ understood the methodology and statistical analysis. JK supervised the project. JF wrote the initial manuscript. All authors contributed to revisions and approved the final version of the manuscript.

## Conflict of Interest

JK has received research support (grants paid to institution) from AbbVie, Amgen, BMS, Boehringer, EMD Serono, Innovaderm, Kineta, LEO Pharma, Novan, Novartis, Paraxel, Pfizer, Regeneron, and Vitae and personal fees from AbbVie, Acros, Allergan, Aurigne, BiogenIdec, Boehringer, Escalier, Janssen, Lilly, Novartis, Pfizer, Roche, and Valeant. The remaining authors declare that the research was conducted in the absence of any commercial or financial relationships that could be construed as a potential conflict of interest.

## Supplementary Material

Supplementary Figure 1 Scatterplots of the relationships between BMI and aspects of disease activity in HS for PIONEER 1 **(A)** and PIONEER 2 **(B)**.

Supplementary Table 1 Table comparing the demographic and disease specific characteristics of participants in PIONEER 1 and PIONEER 2.

Supplementary Table 2 Regression Models with Baseline AN Count as a significant covariate in achieving IHS4 category change (Model 3) and change in AN count (Model 4) in PIONEER 2.
