# Supplementary material for: The Impact of Body Mass Index Upon the Efficacy of Adalimumab in Hidradenitis Suppurativa
Source: Front Med (Lausanne). 2021 Jun 22;8:603281. doi: 10.3389/fmed.2021.603281 (PMC8257943; doi:10.3389/fmed.2021.603281)
Supplement: Supplementary Figure 1 — Scatterplots of the relationships between BMI and aspects of disease activity in HS for PIONEER 1 (A) and PIONEER 2 (B). [file Image_1.pdf]

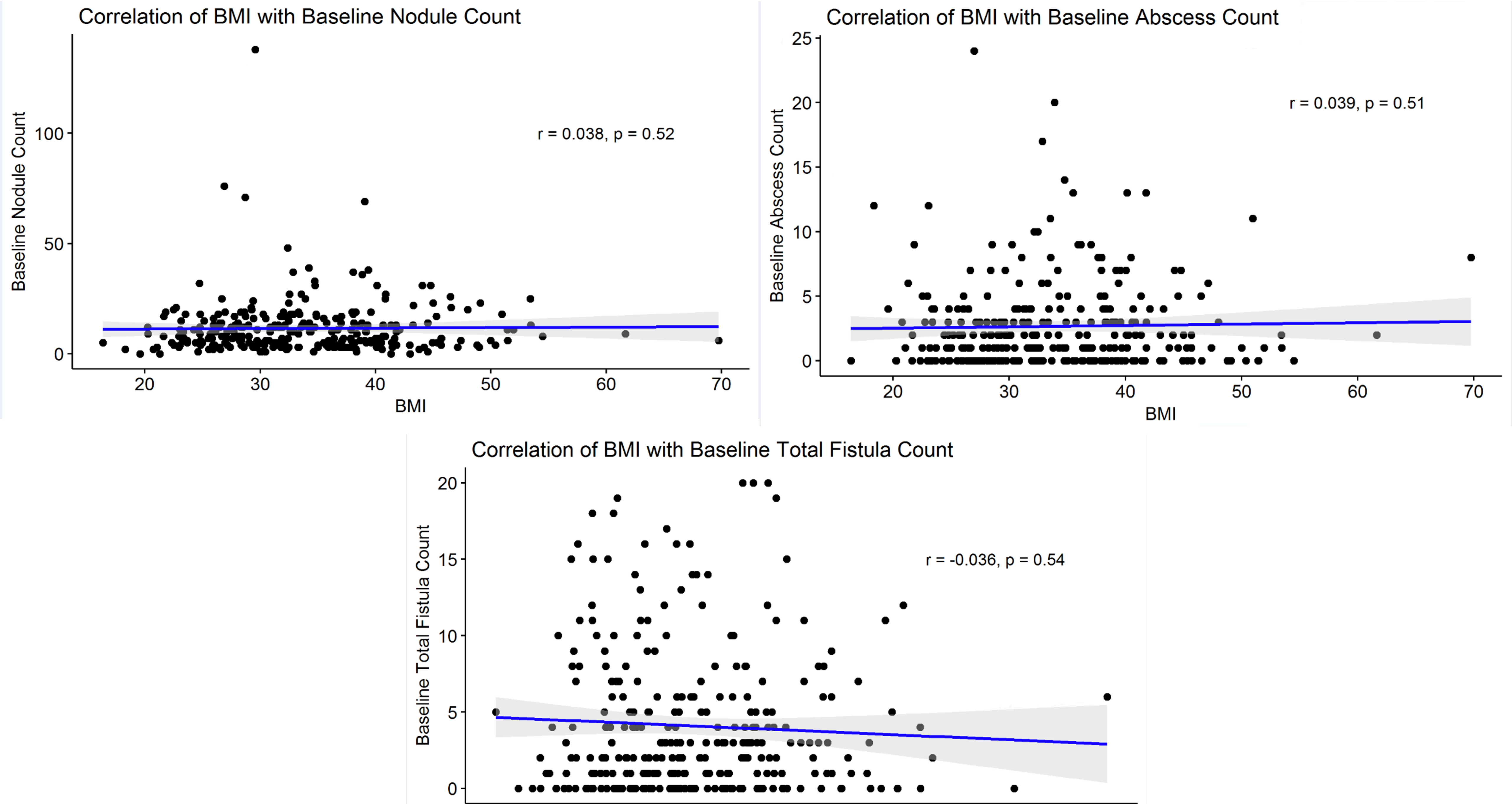
Correlation of BMI with Baseline Nodule Count
r = 0.038, p = 0.52
Baseline Nodule Count
0
50
100
20
30
40
50
60
70
BMI
Correlation of BMI with Baseline Abscess Count
r = 0.039, p = 0.51
Baseline Abscess Count
0
5
10
15
20
25
20
30
40
50
60
70
BMI
Correlation of BMI with Baseline Total Fistula Count
r = -0.036, p = 0.54
Baseline Total Fistula Count
0
5
10
15
20
