# Supplementary material for: The Impact of Body Mass Index Upon the Efficacy of Adalimumab in Hidradenitis Suppurativa
Source: Front Med (Lausanne). 2021 Jun 22;8:603281. doi: 10.3389/fmed.2021.603281 (PMC8257943; doi:10.3389/fmed.2021.603281)
Supplement: Supplementary Table 1 — Table comparing the demographic and disease specific characteristics of participants in PIONEER 1 and PIONEER 2. [file Data_Sheet_1.pdf]

**Characteristic**	**PIONEER 1**	**PIONEER 2**	**P Value**
N	289	289	-
Female	185 (64.0%)	195 (67.5%)	0.43
Male	104 (36.0%)	94 (32.5%)	
**White**	**224 (77.5%)**	**240 (83.0%)**	**0.0003**
**Black**	**55 (19.0%)**	**26 (9.0%)**	
**Other**	**10 (3.5%)**	**23 (8.0%)**	
**Median Age**	**36.0 (29.0, 46.0)**	**35.0 (27.0, 43.0)**	**0.03**
**Median BMI**	**32.5 (28.1, 38.6)**	**31.2 (26.5, 36.0)**	**<0.001**
Hurley 2	159 (55.0%)	155 (53.6%)	0.80
Hurley 3	130 (45.0%)	134 (46.4%)	
**Nicotine Use**	**165 (57.1%)**	**195 (67.5%)**	**0.01**
Family History	65 (22.5%)	75 (26.0%)	0.38
**Presence of Draining Tunnels**	**216 (74.7%)**	**186 (64.4%)**	**0.01**
**Median Nodules**	**8 (4, 14)**	**6 (4, 11)**	**<0.001**
Median Abscesses	2 (0, 4)	1 (0, 3)	0.07
**Median Draining Tunnels**	**2 (0, 6)**	**1 (0, 4)**	**0.002**
**Median Baseline IHS4**	**25 (14, 44)**	**19 (9, 34)**	**<0.0001**

Supplementary Table 1: Comparing Characteristics between PIONEER 1 and PIONEER 2

Table reports N (% in parentheses) with median (25th and 75th percentile) for age, BMI, nodules, abscesses, draining tunnel counts, and baseline IHS4.

Variable	PIONEER 2 Achieving IHS4 Category Change		
Model 3	Odds Ratio	95 % CI	P value
Adalimumab	**2.90**	**(1.73, 4.94)**	**<0.001**
Baseline AN Count	**0.94**	**(0.90, 0.97)**	**0.00**
Hurley Stage 3	0.60	(0.35, 1.02)	0.06
Family History	1.26	(0.70, 2.28)	0.44
Current Smoker	0.96	(0.54, 1.70)	0.88
Antibiotic Use	0.65	(0.33, 1.25)	0.21
BMI (Overweight)	1.49	(0.70, 3.23)	0.30
BMI (Obese)	0.74	(0.38, 1.48)	0.40
Male Sex	0.57	(0.32, 1.00)	0.05
Age	0.99	(0.97, 1.01)	0.37

Variable	PIONEER 2 Change in AN Count		
Model 4	Estimate	95 % CI	P value
Adalimumab	**-2.98**	**(-4.18, -1.78)**	**<0.001**
Baseline AN Count	**-0.37**	**(-0.44,-0.30)**	**<0.001**
Hurley Stage 3	0.38	(-0.88, 1.64)	0.55
Family History	-0.70	(-2.07, 0.67)	0.31
Current Smoker	-0.57	(-1.88, 0.74)	0.39
Presence of Draining Tunnels	**2.50**	**(1.15, 3.84)**	**<0.001**
Antibiotic Use	0.32	(-1.21, 1.85)	0.68
BMI (Overweight)	0.71	(-1.10, 2.51)	0.44
BMI (Obese)	1.26	(-0.34, 2.87)	0.12
Male Sex	-0.22	(-1.57, 1.12)	0.74
Age	-0.01	(-0.06, 0.05)	0.76

Supplementary Table 2: Regression Models with Baseline AN Count as a significant covariate in achieving IHS4 category change (Model 3) and change in AN count (Model 4) in PIONEER 2.
